# Molecular Tissue Responses to Mechanical Loading

**DOI:** 10.3390/ijms23042074

**Published:** 2022-02-14

**Authors:** Joseph P. R. O. Orgel

**Affiliations:** Department of Biology, Department of Physics, Department of Biomedical Engineering, Illinois Institute of Technology, Chicago, IL 60616, USA; orgel@iit.edu

The intention of this special edition is to highlight the benefits of a holistic approach to computational and experimental approaches in the context of aiding the diagnosis and remediation of disease and injury, especially in neurological and connective tissues and organs. The expected benefit is a combined approach that rises above the inherent biases of relying on one approach or technique to explore complex and at times otherwise intractable problems without a clear path to a solution. Then of course as we are all aware, the advent of a ‘100-year’ level disease pandemic descended and disrupted the plans of virtually every person on the planet, including our own. On this point, one of the manuscripts submitted for this special issue contributes to both this combined experimental and computational approach while offering insight into fibrosis and long-term immunological concerns, which has relevance to COVID-19 and long-COVID. The other contributions offer insight into a range of tissue and cellular mechanisms through this experimental and computational approach enabling a view from the nanoscopic to macro-scale.

Practitioners of structural biology are at times overlooked as ‘computational’ specialists. As they are well aware, it is nearly impossible to practice this branch of experimental science without a deep understanding of the benefits and pitfalls of in silico approaches that are not firmly controlled by well-observed data. Further, although it is often stated that computational approaches offer an economy in practice by removing the need for expensive experimentation and real-world applications, the converse can also be true. A well-designed and executed experiment can neatly address a question more readily than conceiving and executing an in silico solution under the right circumstances. In Varma et al 2021 [1], a good correlation was found between experimental observations independent of the collagen structural model and the calculations based on the experimental structure. Specifically that there is little effect on the size and internal structure of the collagen D-band, the unit cell and organizational unit of fibril structure [2]. While at the same time the results indicate a significant drop in inter-monomer hydrogen bonding. Again this is supported by collagen peptide experiments [3,4] that show reduced hydrogen bonding leads to lower melting temperature and that mapping these data onto the helix dissociation of native collagen shows only a loose correlation with macromolecular structure [5], even while the simple assumption is that surely small scale effects over much of a macrostructure should lead to scalable change in that macrostructure. The answer is that depends on the system and its complexity. This consideration has relevance for molecular biology in vivo where peptide dissociation points have some correlation to molecular binding sites of collagen ligands and the complex interplay of the intelligent fiber (collagen) in a living dynamic mechano-chemical semi-aqueous environment [6].

Madhurapantula et al 2020 [7] and Roy et al 2020 [8] were complementary studies designed from their inception to understand a little-explored area of tissue mechanics to enable accurate modeling. In the experimental work, we were able to pinpoint and measure at both macro and nanoscopic scales the most vulnerable points for tissue failure in heart valves. By defining both these points and their material properties, it is envisioned the long-term repair of such tissues, which is ably performed by surgeons today, will further improve with higher quality surgical and regenerative material to properly model that which they replace. In this process, we also have developed the capability to model the effects of global stresses on the cardiovascular system in local regions of high tissue vulnerability.

Much of the work for Zhu et al 2020 [9] was performed over the course of the previous decade. The advent of the coronavirus in early 2020 motivated the completion and publication of this project. This is because we understood the significance of providing what could be pertinent information in the context of the effects of a virus that generates strong immune responses, to collagen. Particularly in the case of the pathological production of collagen, hence the relevance of the molecular interaction of immunological entities with the body’s most prevalent protein, by mass, collagen. Using truly novel antibody-based marking of ex vivo tissue detected by both X-ray diffraction and atomic force microscopy we were able to locate the major histocompatibility complex 1 (MHC1) binding domain. At the same time, this finding clearly answered any remaining question of the exterior of the collagen fibril being lined with the C-terminal end of the collagen monomer, for simple reasons of the accumulated observed structural data and the binding profile of the antibody raised against the C-terminal region. A combination of old workhorse computational methods such as the Patterson function combined with modern molecular modeling and visualization abilities showed close proximity and probably interplay between the MHC1 domain of type I collagen to the binding domains of cell attachment and control regions likely influence one another in questions related to cellular immunity, proliferation, and the extracellular matrix [6,10,11]. This close proximity and likely association of immunological and cellular interactions regions has direct relevance to the remediation of COVID-19 long-term effects during and post-infection. For instance, it was recently reported that the leucine-rich repeat-containing protein 15 (LRRC15) may play a significant role in sequestering viral particles to control infection while also suppressing lung fibrosis [12]. It is remarkable to note, that the apparent structure of LRRC15 may resemble that of other leucine-rich repeat proteins such as decoron (decorin) and biglycan for which structural interactions with the collagen fibril have already been defined through experimental and computational methods [13,14] (Figure 1). Early observations suggest that lung fibroblasts interacting with virus spike+ macrophages and dendritic cells may be assisted by LRRC15. Post inflammation, it appears that LRRC15 may support collagen deposition, where it is dysregulated, leading to inappropriate collagen production and long-term post COVID complications [11,12]. The complex interplay in this most topical of biomedical queries has some direct correlation with the ongoing experimental and computational work in otherwise basic/discovery research. It is earnestly hoped that this work will provide some insight and help in the ongoing efforts to better prevent, diagnose and treat disease and injury.

## Figures and Tables

**Figure 1:**
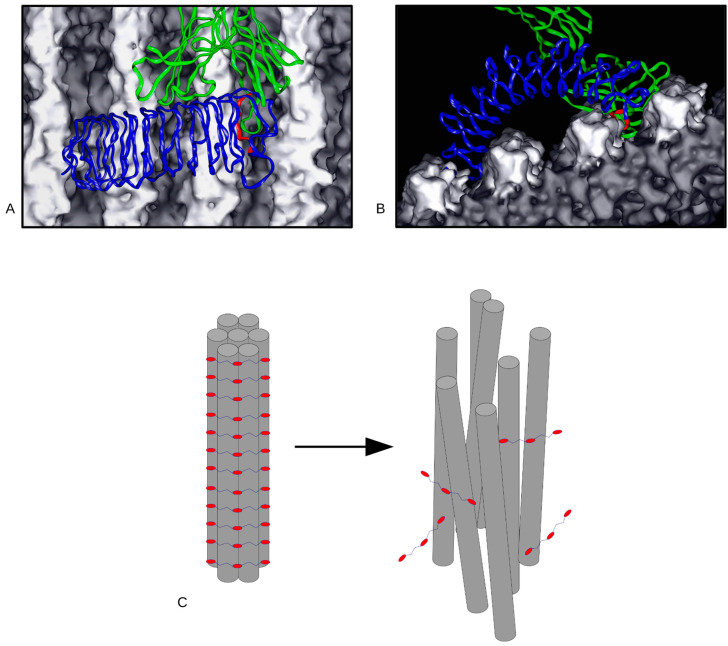
(**A**,**B**) show coordinate models of the interaction of an extracellular matrix LRR proteoglycan (Biglycan, blue), analogous to LRRC15, in its structural role in maintaining fibril-bundle integrity (**C**). Under certain circumstances, antibodies can disrupt this interaction. Antibody fragment binding is shown in (**A**,**B**) and colored green, with a competitive protein-protein interaction between antibody, the LRR protein core (epitope colored red), and fibrils in the fibril bundle (collagen molecules colored light and dark gray). (**C**) shows the disruption to the ECM that occurs under these conditions [14], where proteoglycans are dis-engaged from the fibril bundle, undoing the structure.
